# A Life Threatening Rash, an Unexpected Cause

**DOI:** 10.1155/2014/146251

**Published:** 2014-11-09

**Authors:** Dhiraj Jain, Stalin Viswanathan, Chandramohan Ramasamy

**Affiliations:** ^1^Department of General Medicine, Indira Gandhi Medical College & RI, Pondicherry 605009, India; ^2^Department of Cardiology, Jawaharlal Institute of Postgraduate Medical Education and Research, Pondicherry 605006, India

## Abstract

We describe a 74-year-old man with purpura fulminans and altered sensorium following an acute febrile illness. Intensive sepsis management was to no avail, until institution of doxycycline therapy following confirmation of scrub typhus. Empirical doxycycline needs to be considered in endemic areas for patients presenting with purpura fulminans.

## 1. Introduction

Skin manifestations of rickettsial infections include eschars (single or multiple due to* Orientia tsutsugamushi* and* Rickettsia africae*, resp.) and eruptions which may be either macular, maculopapular, vesicular, or purpuric [1]. Scrub typhus is a ubiquitous infection in the tropics but is usually not considered in the differential diagnosis of life-threatening dermatoses. Rickettsial infections have been, on occasions, reported to cause purpura fulminans. Herein we describe an elderly man with scrub typhus-related purpura fulminans who was cured of his illness, following administration of doxycycline.

## 2. Case

This 74-year-old man presented with continuous fever of five days' duration. Headache, myalgia, and productive cough were also present. There was altered sensorium in the form of decreased speech and irritable behavior one day prior to admission, without vomiting, seizures, or limb weakness. There were no cardiorespiratory, gastrointestinal, or renal complaints. The patient did not have history of diabetes, hypertension, or addiction to either alcohol or tobacco.

**Figure 1:**
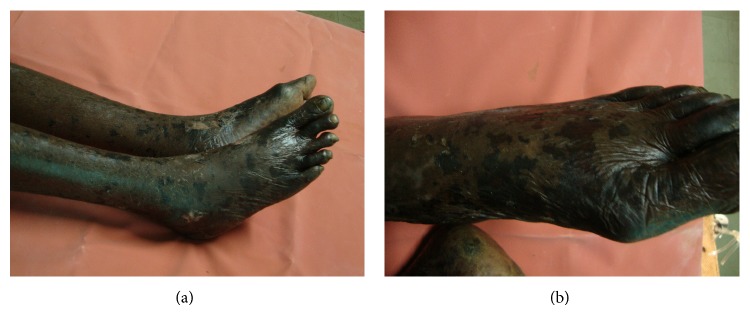
(a) Blotchy purpuric rash over both lower limbs mainly in the extremities, with some healed areas 48 hours after doxycycline therapy. (b) Closer view of left foot showing involvement of all toes and dorsum of foot, with some healed lesions showing hypopigmentation.

On examination, the patient was irritable and disoriented, with a temperature of 38.8°C, pulse of 104 beats/minute, blood pressure of 90/60 mm Hg, and respiratory rate of 24/min. He had pedal edema and an otherwise normal systemic examination. Pending results, ceftriaxone and amikacin were administered for probable sepsis. His investigations were as follows: hemoglobin 12 g%, total leukocyte count 21 × 10^9^/L, associated with left shift and toxic granulations, platelet count 18 × 10^9^/L, blood urea 160 mg/dL, serum creatinine 2.6 mg/dL, INR 1.6, aPTT prolongation 10 seconds, D-dimer 400 ng/mL, total/direct bilirubin 3/2.8 mg/dL, and SGOT/SGPT 73/64 U/L. His blood and urine cultures were noncontributory. Lumbar puncture was deferred in view of severe thrombocytopenia. Computed tomography of head was normal. Following investigations, he was switched to renal-modified doses of ceftazidime. On the second day, he developed a symmetrical purpuric rash involving the palms, soles, arms, and legs which later became bullous, confluent, black, and necrotic over the next 48 hours (Figures 1(a) and 1(b)). Peripheral smear on 3rd day showed features of microangiopathic hemolytic anemia. Serological testing for hepatitis B, hepatitis C, HIV, leptospirosis, scrub typhus, and systemic lupus was ordered. Echocardiography and ultrasonography were noncontributory. Meropenem was substituted for ceftazidime on day 3. On the fourth day, scrub IgM (PanBio, Brisbane, Australia; Scrub Typhus IgM and IgG Rapid Immunochromatographic test) returned positive. Doxycycline was initiated, with which his fever, altered sensorium, and hepatorenal dysfunction improved. His skin lesions resolved without sequelae and he was discharged from hospital on the 7th day of admission.

## 3. Discussion

Purpura fulminans (PF) is a triad of skin ecchymoses, infarction/necrosis, and hemorrhagic bullae which occurs due to both infectious and noninfectious causes [2]. According to one large case series, skin discoloration, disseminated intravascular coagulation, and septic shock were the commonest features of PF [3]. Gangrene occurs distally and is usually symmetrical. This condition is more common in pediatric age groups [4]. Abnormalities can ensue in either the fibrinolytic or coagulation system [4]. Infectious causes of PF include meningococcal (classical) and pneumococcal sepsis and infections due to varicella, influenza, and* Candida albicans* [4]. Congenital and acquired protein C/S deficiencies, antiphospholipid syndrome, paroxysmal nocturnal hemoglobinuria [5], vasculitides, animal/insect toxins, and drugs such as phenytoin, ketorolac, quinidine, and diclofenac contribute to noninfectious PF [2, 4]. Leptospirosis [6], malaria [7], and dengue [8] are some tropical infections reported to have caused PF. Among the rickettsiae,* R. conorii*,* R. rickettsii*, and* R. australis* have produced fatal PF [9, 10]. Antibodies to* R. conorii* were not tested due to its unavailability in our hospital. Indian tick typhus has been described as an etiological factor for PF from various parts of India [11–13]. But scrub typhus has not been previously reported to cause PF. Infectious PF usually develops 1–3 weeks after the infective episode [4].

Management of PF requires a multidepartment effort that involves physicians/pediatricians, surgeons, radiologists, nurses, and physiotherapists. Interventions such as debridement, fasciotomy, amputations, and thrombolysis may be sometimes necessary [3, 14]. For compartment syndromes arising due to PF, relief should be provided within six hours. Medical therapy comprises antibiotics, steroids, and protein C infusion [15] along with routine sepsis care. Our patient did not require any surgical intervention and improved with doxycycline alone.

In conclusion, PF is a potentially fatal dermatological emergency that requires intensive and multidisciplinary care. Purpura fulminans may be treated more appropriately by familiarizing oneself with prevalence of illnesses in the locality, which could reduce morbidity, mortality, and hospitalization stay. Although scrub typhus has been widely reported from many states in India, the exact prevalence is not known. Though Indian tick typhus much more commonly causes rickettsia-related purpuric eruptions, scrub typhus, by virtue of its prevalence in the tsutsugamushi triangle, should not be forgotten in the Indian subcontinent.
